# A national quality assurance programme for point-of-care testing in Malawi

**DOI:** 10.4102/ajlm.v5i2.540

**Published:** 2016-10-17

**Authors:** Lutho I. Zungu, Termson Magombo, Tarsizio Chikaonda, Rachel Thomas, Reuben Mwenda, James Kandulu, Benson Chilima, Mavuto Chiwaula, Alwin Mbene, Emmanuel Saka

**Affiliations:** 1 Ministry of Health Directorate of Health Technical services – Diagnostics, Lilongwe, Malawi; 2 Clinton Health Access Initiative, Lilongwe, Malawi; 3 University of North Carolina (UNC) HIV/AIDS Research Project Laboratory, Lilongwe, Malawi; 4 National Reference Laboratories, Community Health Sciences Unit (CHSU), Lilongwe, Malawi; 5 Northern Health Zone, Ministry of Health, Lilongwe, Malawi; 6 UNICEF, Lilongwe, Malawi

## Country situation, including HIV status

Malawi is a landlocked country, stretching over 94 084 square kilometres of land and 24 404 square kilometres of fresh water, whose economic backbone is agriculture. Malawi’s HIV positivity rate is estimated at 10.0% (Table 1).^1^ HIV prevalence varies widely by geographic regions, where it is 14.5% in southern Malawi, 7.6% in central Malawi and 6.6% in northern Malawi. Prevalence also differs by level of urbanisation in line with population density. Rural Malawi has a positivity rate of 8.9%, which is half the rate of the 17.4% found in urban areas.^1^

**TABLE 1:** Malawi key statistics: Brief demographic summary.

Key statistics	Value	Key statistics	Value
Total population	17 964 697†	People living with HIV in Malawi	1 100 000‡
Birth rate (births/1000 population)	41.56	Total people alive and on ART by end June 2015	568 470
Death rate (deaths/1000 population)	8.41	Infants receiving a timely (< 2 months of birth) virological test	54%
Maternal mortality rate (deaths/100 000 live births)	634	Number of health facilities offering ART services	711
Life expectancy males (years)	58.67	Number of health facilities offering PMTCT services	601
Life expectancy females (years)	62.69	Number of health facilities offering EID services	642
Adult HIV prevalence rate	10%	Number of health facilities offering HIV testing	850

† National Statistical Office 2008 Population and Housing Census;

‡ 2014 National AIDS Commission projections.

ART, antiretroviral therapy; PMTCT, prevention of mother-to-child transmission; EID, early infant diagnosis.

## Laboratory infrastructure and HIV related testing in Malawi

**FIGURE 1:**
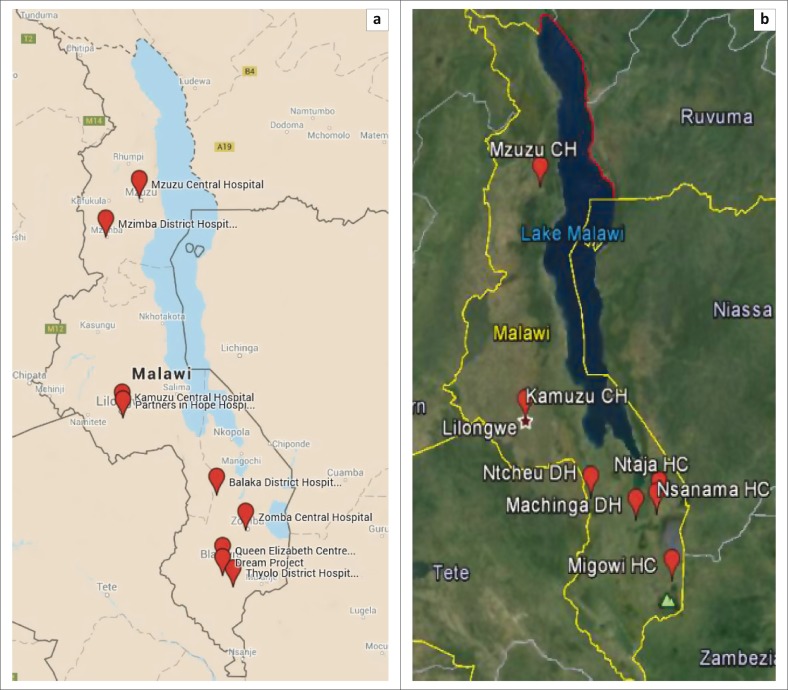
Distribution of PCR laboratories and early infant diagnosis point-of-care testing in Malawi. (a) Red balloons show the nine conventional PCR laboratories. (b) Red balloons show the seven pilot sites for early infant diagnosis point-of-care testing.

Malawi has nine molecular laboratories that serve as hubs for PCR testing and process all HIV viral load and early infant diagnosis (EID) samples collected on dried blood spots (Figure 1). Malawi is piloting point-of-care (POC) testing for EID. Seven health facilities are participating in this pilot. Table 2 shows the number of CD4 testing devices at facilities at different levels of the public health care system, as well as at partner and private laboratories.

**TABLE 2:** Distribution of laboratory and point-of-care CD4 testing in Malawi

Device Name	Central Hospital	CHAM Facility	Rural Hospital	District Hospital	Health Centre	Private Hospital	Partner Facility	Total
BD FacsCount	6	10	5	26	4	1	3	55
Partec CyFlow	2	7		21			1	31
Pima Analyser		6	7	3	111	6	1	134
Partec CyFlow and Pima	3	-	-	3	-	-	-	6
Total Devices	11	23	12	53	115	7	4	226

CHAM, Christian Health Association of Malawi.

Table 3 shows the number of CD4, HIV EID and viral load tests performed on both laboratory and POC platforms. The low percentages for the targets in all categories demonstrate that there is a large gap between demand and needs met.

**TABLE 3:** Summary of CD4, early infant diagnosis, and viral load testing in 2015.

Type of test	Total needed	Total tests performed	Percentage of target
CD4 testing	312 012	90 215	29%
Conventional laboratories	-	40 000†	-
Point-of-care	-	50 215	-
Early infant diagnosis	57 000	34 805	61%
Conventional laboratories	-	34 305	-
Point-of-care (piloting)	-	500	-
Viral load testing	415 112	108 821	26%
Conventional laboratories	-	108 821	-
Point-of-care (N/A)	-	0	-

† The lower uptake on conventional platforms was due to reagent stock-outs; N/A, not applicable.

Besides the ongoing implementation pilot of POC testing for EID in tertiary, secondary and primary care settings, Malawi is also considering POC testing for HIV viral load in 2016 and 2017 on the most relevant platforms available on the market.

## Malawi’s quality assurance framework and policy for HIV laboratory and point-of-care testing

POC testing has a significant part to play in the delivery of efficient healthcare services, as rapid availability of test results can lead to increased clinical effectiveness, less loss to follow-up and improved outcomes for patients. POC testing has also helped expand laboratory services to hard-to-reach areas that lack trained technicians or constant electricity supply.^2^ However, along with the scale-up of POC/near-POC diagnostics, there is a critical need to ensure the quality and accuracy of testing results through proper training and innovative quality assurance activities.

A formal policy defining the principal role of the diagnostic division of the Health Technical Services Directorate in Malawi has become indispensable. This ensures that the whole process of testing is conducted in accordance with the fundamental principles of clinical governance and national, as well as international, accreditation standards to improve and sustain quality.

Currently, the professional partnership that exists between the diagnostics division, clinical, and implementing partners in Malawi ensures that POC testing equipment is suitable for its intended use, is adequately supported (in terms of consumables and maintenance), safety and quality standards are adequately met, results of investigations performed are recorded, and that it is operated only by well-trained staff. The Diagnostic Technical Working Group has established a POC testing subcommittee to provide technical advice about equipment and consumables, user training, internal quality assurance and external quality assurance (EQA) implementation, support and accreditation.

To systematically guide this process, the following documents have been developed:

- POC guidelines.
- Sample transportation guidelines.
- HIV Testing and Counseling guidelines.
- GeneXpert guidelines.
- Quality assurance manual.
- HIV clinical management guidelines for adults and children.
- Procurement guidelines.
- Training certification guidelines.

To monitor and improve the quality and accuracy of test results, various tools have been developed and are now being implemented as part of the quality framework. These tools include the following:

- The dried tube specimen-based national proficiency testing programme.
- Standardised HIV logbooks for national quality assurance programme.
- HIV rapid testing quality improvement initiative.
- Registration for enrollment on international EQA schemes.

**FIGURE 2:**
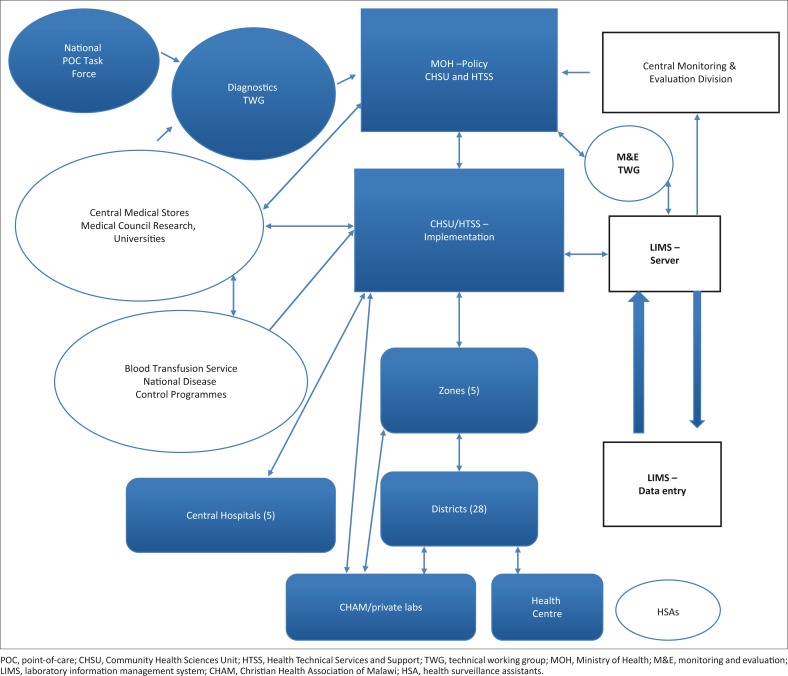
Malawi’s centralised quality assurance framework.

This quality framework is summarised in Figure 2.

The central level includes Community Health Sciences Unit and Health Technical Services and Support -Diagnostics. Together with partners, the central level is responsible for: preparation of quality control materials; identifying and correcting problems; evaluating kits and reagents; standardisation of procedures and developing standard operating procedures; preparing reagents; training; collating reports; analysing data; offering feedback; conducting disease surveillance; and evaluating and standardising equipment. The National POC testing Task Force (subcommittee) reports to National Diagnostics Technical Working Group, which in turn reports to the Ministry of Health. Zones hold a support/coordination role. At the zonal level (5 Zones), laboratory supervisors follow quality assurance issues, including supervision, mentorship, training, EQA, corrective actions, etcetera. District laboratories supervise the health centres and follow up on quality assurance issues, in the same manner as the laboratory supervisors.

## Existing quality assurance programmes and lessons learnt

Currently EQA schemes (both international and national) to which testing sites are registered and subscribed include the following:

- **National Health Laboratory Services:** This is a South African scheme that provides proficiency testing samples in chemistry, haematology, and cell morphology (monthly). In parasitology, samples for blood and stool parasites are provided three times a year. For tuberculosis microscopy, quality control materials are provided three times a year, whereas for bacteriology cultures, the scheme sends EQA panels three times a year.
- **UKNEQAS:** The scheme sends EQA panels three times per year in the areas of CD4, tuberculosis microbiology, haematology and chemistry.
- **AFRIQUALAB:** Under this scheme, the samples are provided three times a year, for CD4, tuberculosis microbiology, haematology, chemistry and HIV.
- **QASI, Canada:** This scheme provides free proficiency testing panels three times a year for approximately 60% of CD4 POC testing sites. However, current efforts to scale the scheme up to 100% have slowed down because Malawi has changed direction in order to adopt a universal testing and treat policy during the second quarter of 2016.

At the local level, there are also national quality assurance schemes organised and provided by the Public Health and Reference Laboratory:

- HIV proficiency testing (biannual).
- Malawi Blood Transfusion Services.
- Central Reference Laboratory for tuberculosis microscopy and GeneXpert^®^; and
- Microbiology quality control materials.

### Lessons learnt and challenges

Training of POC testers to understand the need for quality assurance is key. Where implementation is coordinated multilaterally (donors, partners, and government functionaries), great success is achieved, for instance in CD4 and viral load testing implementation. In addition, sample transportation systems are of great importance, which are being conducted by Riders for Health in Malawi.

Participation in EQA schemes is improving, even though some participating laboratories/sites delay submission of EQA results. Inadequate knowledge of some testers with respect to interpretation of EQA results is an issue in some schemes. Where schemes are not well understood, some laboratories are enrolled in two or three schemes for the same assay, yet have limited interest in performing EQA testing.

Inconsistent supply of laboratory reagents due to stock outs or poor management of the supply chain has negatively impacted EQA implementation. In addition, long periods of equipment breakdown also negatively impact EQA.

Some further challenges are that not all tests have been enrolled in EQA schemes, which is often due to the cost of enrollment. The lack of availability of internet connectivity in many sites also hinders success of EQA schemes. Finally, the existence of vertical disease control programmes compromises collaborative efforts aimed at leveraging resources to maximise productivity and achievement.

### National quality assurance programme for point-of-care testing in Malawi

**FIGURE 3:**
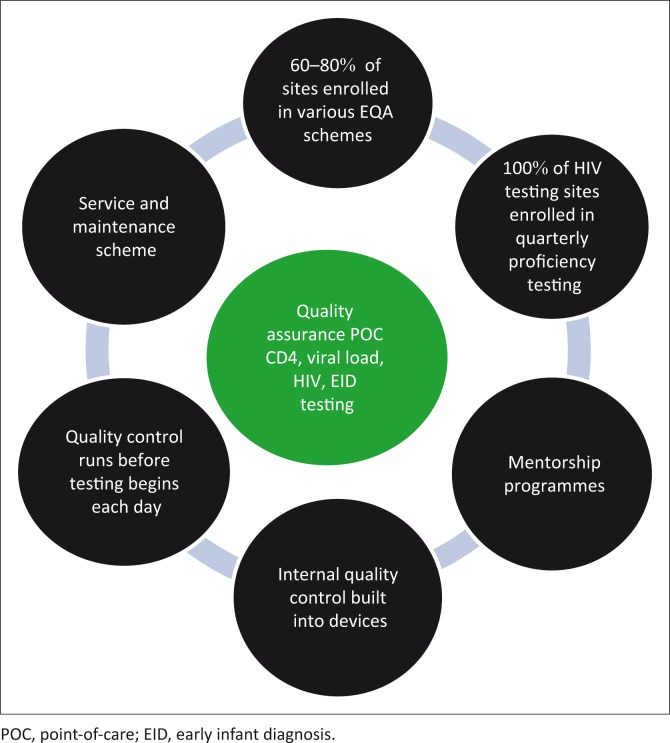
Schematic diagram of Malawi’s point-of-care testing testing quality assurance operational framework showing components that support the quality assurance programme.

While the principles of quality assurance are the same for POC testing and conventional, laboratory-based testing, the methods by which they are applied differ, depending on many factors. These factors may include clinical setting, testing frequency, complexity of the device and internal controls, cost and practicality of providing the quality assurance system, and whether laboratory or non-laboratory end users are the ones operating the devices.^3^ Currently, the Malawi POC testing QA framework follows the structure of the national QA framework (Figure 3).

Manufacturers have recognised that the main consumers of POC tests are healthcare workers with non-laboratory backgrounds. Hence, POC testing units have been developed with built-in quality control checks and connectivity to allow for real-time oversight of testing.

EQA programmes are a component of a continuous quality assurance and improvement cycle, and under International Organization for Standardization (ISO) standards, are a mandatory requirement for medical laboratories.^4,5^ The requirement is reflected in the international standard, ISO 22870 POCT – requirements for quality and competence.^5^

### Conclusion

Malawi has thus far provided reasonable coverage for EID and viral load testing through its corridor of nine molecular laboratories that serve as hubs. The hub system and dried blood spots have allowed for increased coverage. Malawi is currently piloting POC testing for EID to reach even more remote settings.

The professional partnerships that Malawi Ministry of Health has fostered between the diagnostics division, clinical, and implementing partners has supported the necessary guidance that will allow for continuous monitoring and further improvements. This partnership has been realised with the POC task force. In addition, a formal POC policy defining the principal roles of partners has become indispensable.

Similarly, Malawi has various partnerships with numerous EQA providers to support quality testing. While participation in EQA schemes is improving, we are looking to use the results from EQA to identify and improve on the inconsistent supply of laboratory reagents due to stock-outs or poor management of the supply chain.
